# Chronic Microvascular Complications in Prediabetic States—An Overview

**DOI:** 10.3390/jcm9103289

**Published:** 2020-10-13

**Authors:** Angelika Baranowska-Jurkun, Wojciech Matuszewski, Elżbieta Bandurska-Stankiewicz

**Affiliations:** Clinic of Endocrinology, Diabetology and Internal Medicine, Department of Internal Medicine, School of Medicine, Collegium Medicum, University of Warmia and Mazury, Żołnierska 18, 10-561 Olsztyn, Poland; wmatuszewski82@wp.pl (W.M.); bandurska.endo@gmail.com (E.B.-S.)

**Keywords:** prediabetes, diabetes, microvascular complications

## Abstract

A prediabetic state is a major risk factor for the development of diabetes, and, because of an identical pathophysiological background of both conditions, their prevalence increases parallelly and equally fast. Long-term hyperglycemia is the main cause inducing chronic complications of diabetes, yet the range of glucose levels at which they start has not been yet unequivocally determined. The current data show that chronic microvascular complications of diabetes can be observed in patients with abnormal glucose metabolism in whom glycaemia is higher than optimal but below diagnostic criteria for diabetes. Prediabetes is a heterogenous nosological unit in which particular types are differently characterized and show different correlations with particular kinds of complications. Analysis of the latest research results shows the need to continue studies in a larger population and can imply the need to verify the currently employed criteria of diagnosing diabetes and chronic complications of diabetes in people with prediabetes.

## 1. Definition and Prevalence

Diabetes (diabetes mellitus, DM) comprises a group of metabolic disorders characterized by hyperglycemia resulting from disorders in insulin secretion or action. Chronic hyperglycemia in DM leads to damage, impaired function or insufficiency of various organs, especially eyes, kidneys, nerves, the heart, and blood vessels [1]. There are 463 million diabetes patients, that is 9.3% of the world’s population. It is estimated that, in 2045, there will be 700 million patients (10%) [2].

Prediabetes is the main risk factor for DM. Analogically to the increase of DM prevalence, due to their identical pathophysiological background [3], the prevalence of prediabetes also increases. Prediabetes as a separate nosological unit is characterized by impaired glucose metabolism with glycemia above the optimal value but still below the diagnostic levels for DM.

Complete epidemiological data concerning prediabetes are not available as there are no uniform diagnostic criteria that would be used by all scientific societies (Table 1), and there are no representative data from most of the world’s countries [2,4,5].

In the USA, the National Examination of Health and Nutrition Survey (NHANES), in the years 1999–2002 and 2007–2010, reported an increase in the prevalence of prediabetes, which was higher in women than men; yet prediabetes, as such, appeared more often in men. Interestingly, the prevalence of impaired fasting glycemia (IFG) remained quite stable—increasing from 23.9% to 26.6.%, contrary to the criteria which employ glycated hemoglobin (HbA1c) (American Diabetes Association criteria), according to which the prevalence increased from 9.5% to 17.8% [14]. It is estimated that, in 2018, 33.9% (48.3% people above 65 years of age) adult Americans age 18 or older (88 million) suffered from prediabetes, evaluated on the basis of fasting plasma glucose (FPG) or percentage of HbA1c [15]. The global prevalence of IFG is estimated to be 5%, yet one needs to remember that there are two criteria of diagnosing IFG used [16].

**Figure 1:**
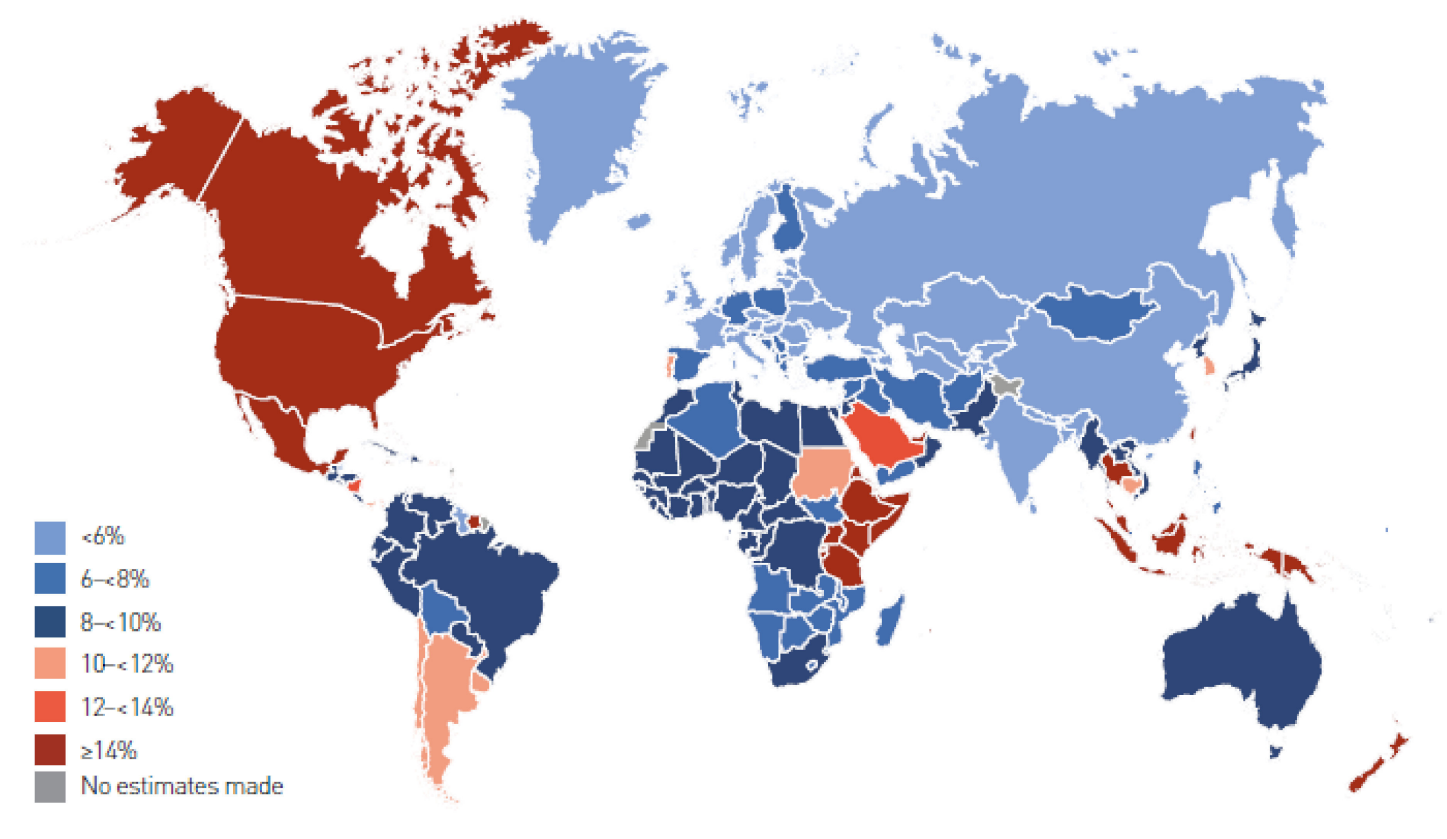
Age-adjusted comparative prevalence of impaired glucose tolerance in adults (20–79 years) in 2019, adapted from International Diabetes Federation. IDF Diabetes Atlas, 9th edn. Brussels, Belgium [2].

Uniform diagnostic criteria of impaired glucose tolerance (IGT) make it possible to compare its prevalence all over the world (Figure 1). In 2019, almost 374 million (7.5%) people in the world were said to have IGT, while it is estimated that, in 2030, this will be 453 million (8.0%), and, in 2045—548 million (8.6%). It is surmised that, in 2030, prediabetes will concern more than 470 million people all over the world [17].

Long-term hyperglycemia is the main cause of chronic complications of DM, which are divided into microvascular (diabetic kidney disease, diabetic retinopathy, and diabetic neuropathy) [18] and macrovascular (ischemic disease of the heart, brain and lower limbs) [19]. There is a “gap” in the glycemic status between correct values and DM. The current review shows that, in many patients, those glucose levels which are between optimal and indicative of DM can also induce the development of chronic microvascular complications. Prediabetes is a heterogenous nosological unit in which particular types are differently characterized and show different correlations with particular kinds of complications. The explanation of the relationship between prediabetes and chronic microvascular diabetes complications is clinically important, as well as important to public health, as early diagnosis of the disorder could be the basis for early therapeutic interventions that could prevent progression of complications or even cause their resolution.

## 2. Methodology

The aim of the study was to discuss the occurrence of chronic microvascular complications of diabetes in prediabetic states.

### 2.1. Pathophysiology

#### 2.1.1. From Correct Glycaemia Through Prediabetes to Diabetes

In healthy people, the blood glucose level is precisely regulated. FPG usually remains at the level 3.9–5.6 mmol/L (70–101 mg/dL) [20]. All over the world, it was observed that the mean FPG increases. In 2008, the mean world standardized FPG was 5.5 mmol/L (99 mg/dL) in men, was 5.42 mmol/L (97 mg/dL) in women, and increased by 0.07 mmol/L and 0.09 mmol/L per decade, respectively [21]. After a meal, the increases infrequently exceed 3 mmol/L (54 mg/dL) [22]. During the development of diabetes mellitus type 2 (DM2), the homeostasis of FPG and post-load glycaemia becomes disrupted [23].

**Scheme 1:**
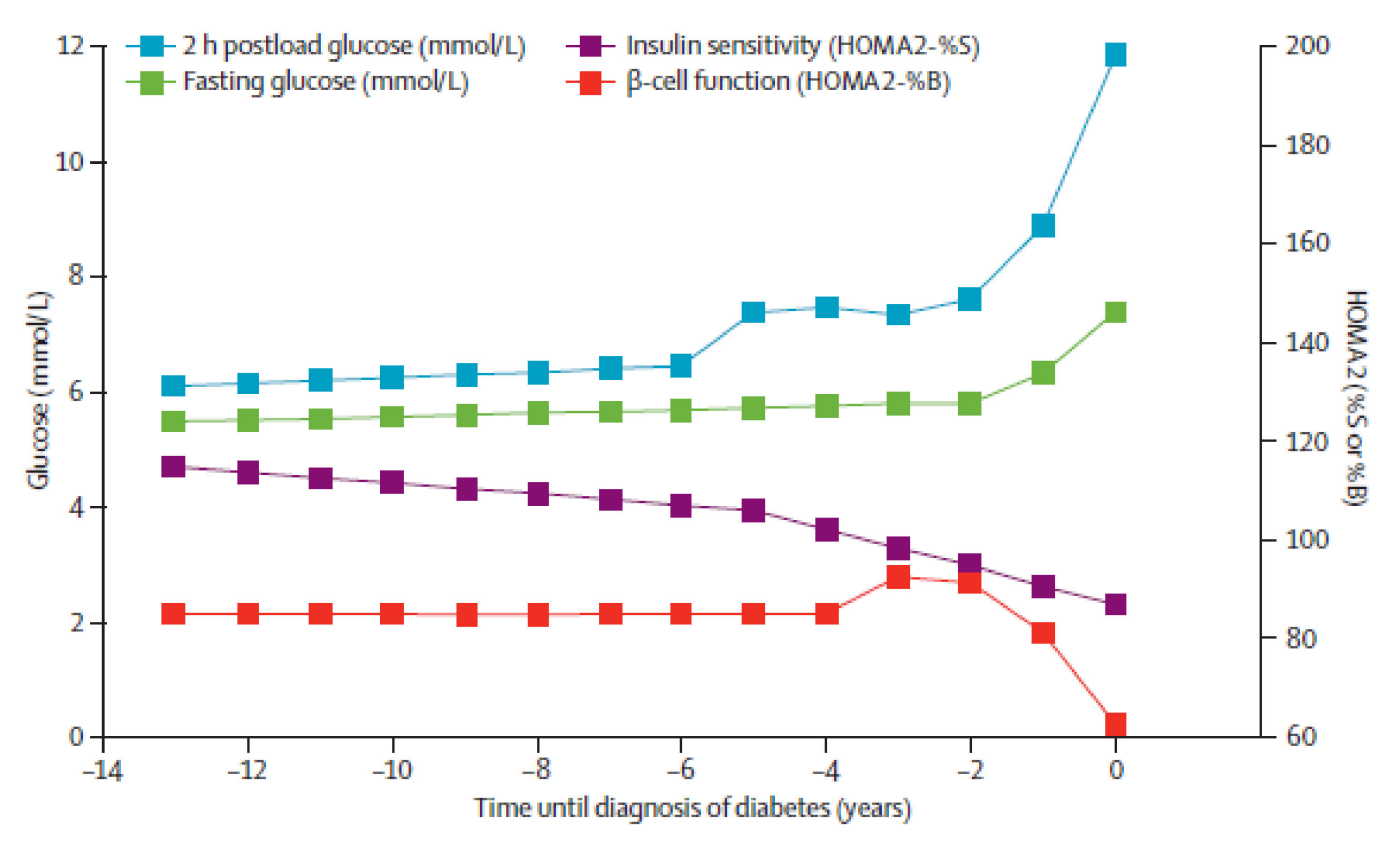
Fasting and 2-h post-load glucose, insulin sensitivity, and β-cell function trajectories before the diagnosis of diabetes, adapted from Tabák et al. [17].

As was proven in studies analyzing dependencies of repeated tests of glucose levels with insulin secretion and sensitivity to insulin, the development of diabetes is a continuous process. In the Whitehall II study, in people who developed diabetes, increased glucose levels were observed as early as 13 years before the diagnosis, although glucose values seemed precisely regulated within the correct range up to 2–6 years before the diagnosis, until their sudden increase [24]. This was also confirmed in other studies [25,26,27]. Sensitivity to insulin was already lowered 13 years before the onset of DM, while its steep decrease occurred 5 years before the diagnosis. Insulin secretion (β-cell function) remained at the same level for 13 years during the observation period, 3–4 years before the diagnosis there occurred a compensational increase of insulin secretion, while immediately before the diagnosis there was a sudden decrease [23]. These results confirm the hypothesis that insulin resistance emerges a number of years before DM, and the reduction of β–cell function appears already in a prediabetic state [20,28] (Scheme 1).

In addition, Weir presented a multi-stage model of diabetes development [29]. The first stage consists in a long period of insulin resistance which is compensated for by increased secretion of insulin and increased β-cells mass [23]. The second stage brings a stable period of adaptation when β-cells gradually cease to be able to compensate for the increased insulin resistance. This period probably starts when FPG and post-load glycemia are still within norm but not optimal [23,24,29]. A number of abnormalities of the first and second stage occur, thus, before the prediabetic stage. During the early stage of decompensation—i.e., the third stage of DM development—β-cells become incapable of compensating for insulin resistance by secreting more insulin, and the glucose level rapidly increases [23,29]. This period probably starts with the prediabetic state and lasts until the manifestation of DM [29].

#### 2.1.2. IFG and/vs. IGT

People with IFG and IGT manifest abnormalities characteristic of DM2, that is, insulin resistance and β-cells dysfunction [30]. However, patients with isolated IFG differ from patients with isolated IGT by their fasting and 2-h post-load glucose levels and the shape of the curves for glucose levels during oral glucose tolerance test (OGTT). Both groups show resistance to insulin, but the places of abnormalities are different.

FPG values are determined by endogenous glucose production (EGP), which depends mainly on the liver. EGP and the fasting insulin level are employed as insulin resistance markers for the liver and show a strong correlation with FPG [22,23,31]. Early (0–30 min) insulin response during OGTT and insulin secretion in the first phase (0–10 min) during the hyperglycemic clamp and intravenous glucose tolerance test are disturbed in IFG [32]. When the FPG level is above 110 mg/dL (6.1 mmol), insulin secretion in the first phase is heavily impaired. However, late (60–120 min) insulin response during OGTT and the second phase of insulin secretion during the hyperglycemic clamp are within range. The hyperinsulinemic euglycemic clamp was used to perform quantitative assessment of glucose uptake dependent on insulin (it first of all reflected muscle uptake), in subjects with IFG insulin sensitivity, was assessed as correct/almost correct. Despite an initially incorrectly high (0–60 min) increase of plasma glucose level after ingesting glucose, the correct sensitivity of muscles to insulin and the correct second phase of insulin secretion allowed 2-h post-load plasma glucose levels in subjects with IFG to come back to correct values (<140 mg/dl, (7.8 mmol/L)). The reason behind increased EPG in subjects with IFG is not entirely comprehensible. It was proved that the basal glucose production rate in the liver (which is the main indicator of FGP level in DM2 patients) is lowered in people with IFG, in comparison with subjects with normal glucose tolerance (NGT). This observation indicates that a decrease in the glucose clearance rate is the most probable reason behind an increased value of FPG in IFG [33]. Summarizing, IFG is characterized by an increased FPG level, high insulin resistance of the liver, impaired early insulin response, excessive early increase (0–60 min) of plasma glucose level during OGTT, and correct 2-h post-load plasma glucose level, as well as correct insulin sensitivity in skeletal muscles [22,23,31].

At the same time, tests with the hyperinsulinemic euglycemic clamp in patients with IGT showed impaired activity of insulin in reducing glucose levels in the entire body (mainly in muscles). Late insulin response (60–120 min) during OGTT and insulin secretion in the second phase during the hyperglycemic clamp are heavily impaired in subjects with IGT [33]. Concluding, in people with IGT, FPG is correct with small changes of insulin sensitivity in the liver, and early and late phase of insulin secretion is impaired [21,22,31], while the main place of insulin resistance occurs in the muscles [20,22].

The above descriptions show considerable differences in the pathophysiological mechanisms of isolated IFG and isolated IGT, while the clinical significance of these disruptions requires further investigation.

#### 2.1.3. The Mechanism Behind Complications

**Figure 2:**
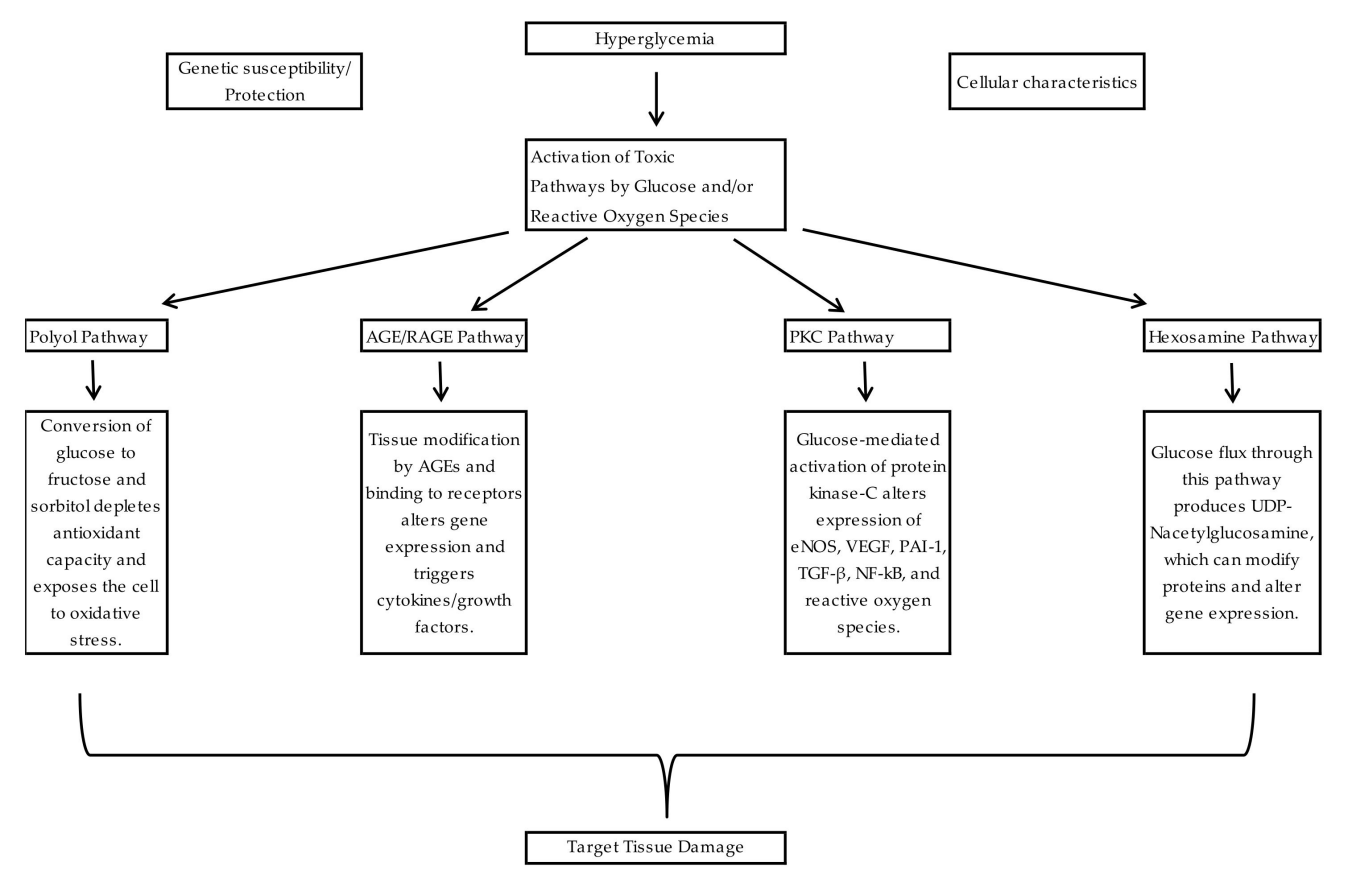
Toxic pathways linking blood glucose levels to tissue damage in susceptible persons, adapted from Brannick et al. [3]; AGE, advanced glycosylation end products; eNOS, endothelial nitric oxide synthase; NF-κβ, nuclear factor-κβ; PAI-1, plasminogen activator inhibitor-1; PKC, protein kinase C; RAGE, receptor for AGE; TGF- β, transforming growth factor-β; UDP, uridine diphosphate; VEGF, vascular endothelial growth factor.

Pathogenesis of microvascular complications in people with glucose metabolism disorders has not been fully explained yet. Apart from genetic predispositions, some of the proposed mechanisms encompass hyperglycemia-induced changes in the pathway of polyol, hexosamine and protein kinase C (PKC), advanced glocalization of proteins, glomerulus filtration, inducing the production of transformation growth factor (TGF-β), and other harmful growth factors, as well as oxidative stress [3]. Among various factors, it seems that these are hyperglycemia-related mechanisms which have a particularly significant effect on the development of the analyzed complications. Intracellular hyperglycemia was found to be related with activation of four toxic pathways which can lead to damage in tissues: increased inflow through the polyol pathway, creating advanced final products of glycosylation, increased activity of the hexosamine pathway and activation of PKC (Figure 2) [34]. Action through those toxic pathways was documented on the basis of experimental models, as well as in people with DM. However, clinical experience shows that actual damage of tissues leading to complications requires a few years of exposure to uncontrolled glycemia. Thus, it is unclear why some people with glycemia below the diagnostic criteria for DM become prone to “premature” development of complications usually observed in patients with long-term DM. It is possible that particular people differ in their response to various levels of glycaemia in relation to the thresholds triggering these toxic pathways. Another possible option is the multiplication effect, in which simultaneous activation of many mechanisms may make some people especially prone to premature damage [3].

In fact, one toxic pathway including PKC activation combines together an increased level of blood glucose with inducing mechanisms triggering tandem changes in expression of nitric oxide synthase (NOS), vascular epithelial growth factor (VEGF), plasminogen-1 activator inhibitor (PAI-1), transformation growth factor β (TGF-β), reactive forms of oxygen, and nucleus kappa-β factor (NF-κβ) (Figure 2) [34]. Effects of glycemic PKC activation, especially those encompassing vascular and inflammatory pathways, can be the cause of impaired β-cells function, insulin resistance, and microvascular and macrovascular complications in susceptible persons. Thus, the current knowledge suggests that moderately increased blood glucose levels in prediabetes can exert negative effect in susceptible persons [3].

### 2.2. Microvascular Complications

#### 2.2.1. Diabetic Retinopathy

A number of studies confirmed a connection between prediabetes and diabetic retinopathy (DR), yet their conclusions are rather diversified, depending on the employed methods [35]. It is estimated that DR can occur in 8–12% of people with prediabetes. In the Diabetes Prevention Program (DPP), DR was found in 7.9% participants with IGT [36]. The connection between IGT and DR was confirmed in the Funagata study, yet the correlation between IFG and DR was not statistically significant [37]. A similar prevalence of DR (8.1%) was found to occur in people with prediabetes in the Gutenberg Health Study [38]. In the AusDiab study, symptoms of DR were diagnosed in 6.7% of people with IFG and/or IGT [39]. A Swedish study carried out among patients with FPG disorders found symptoms characteristic of DR in 10% [40]. Moreover, studies using confocal microscopy and skin biopsy detect small-fiber neuropathy in patients with IGT [41].

In the analysis of three big studies scrutinizing the effect of FPG levels increasing to DM diagnostic values (126 mg/dL) on DR occurrence, it was determined that there is a direct correlation, yet no cohesive and clear threshold, for glycaemia at which DR appears was found [39]. Assessing the connection of DR prevalence with HbA1c and FPG in the population above 40 years of age in the USA, a rapid growth of DR was detected, when HbA1c exceeded 5.5%, and FPG exceeded 104.4 mg/dL, while HbA1c was a better predicative factor than FPG [42]. In a 2019 study among patients with prediabetes (IFG + HbA1c 5.7–6.4%), DR was diagnosed in 24.1% of the group. The percentage of people with DR increased linearly together with the increasing percentage of HbA1c, respectively: HbA1c < 5.7%: DR 11.4%, HbA1c 5.7–6.0%: DR 23%, HbA1c 6.0–6.4%: DR 28.1% [43].

The studies suggest that the effect of glucose and blood pressure on microvascular circulation in the retina is gradual and continuous, and the currently applied definitions of diabetic and hypertensive retinopathy are arbitrary and do not focus on the early stages of the diseases [44]. There are attempts made to assess early symptoms of secondary arteries dysfunction of retina in prediabetes with the use of advanced tools, which cannot be detected in a routine clinical study [45].

#### 2.2.2. Diabetic Kidney Disease

For some years, evidence for a correlation between an increased risk of chronic kidney disease (CKD) and diabetic kidney disease (DKD) in prediabetes has been recorded [35,46]. Characteristic features found in 14 cohort studies are summarized in Table 2 [47,48,49,50,51,52,53,54,55,56,57,58,59,60]. Half of the studies [48,49,50,51,52,53,54] were designed mainly to assess the relationship between metabolic syndrome and CKD. The size of groups in the presented cohorts ranged from 2398 to 118,924. The European population [55,56,58] and the American population [47,48,50] were each assessed in three studies, while eight studies concerned the Asian population [49,51,52,53,54,57,59,60]. Some of the European and American studies included multi-ethnic populations, but the white population constituted at least 50% in each of them. One of the studies concerned only men [52], while others analyzed both sexes. The age of the subjects ranged from 18 to 84 years. The period of observation ranged from 2 to 9 years, with the mean value of 5.5 years. The definition of prediabetes varied in the studies: eight studies considered only IFG, and IFG + IGT, IFG + HbA1c, and IFG + IGT + HbA1c combinations were considered in two studies each. In nine studies, IFG was defined as fasting glucose between 110 and 125 mg/dL; in four studies, this was 100–125 mg/dL, while, in one study, two ranges were taken into consideration [57]. In all the studies scrutinized here, people with diabetes were excluded from participation in the research. In eight studies, estimated glomerular filtration rate (eGFR) < 60 mL/min per 1.73 m^2^ was assumed when defining CKD in line with Modification of Diet in Renal Disease (MDRD); in four studies, Chronic Kidney Disease Epidemiology Collaboration (CKD-EPI) was used, and single studies employed Cockroft–Gault equation [50], or isotope dilution mass spectrometry traceable 4-variable Modification of Diet in Renal Disease (IDMS-MDRD study equation) [57]. Five studies additionally included microalbuminuria or proteinuria in the definition of CKD [49,50,53,57,58]. The degree to which the co-variables were adapted differed in particular studies; only six studies included the baseline eGFR [47,52,55,57,59,60]. Only in four out of fourteen studies did IFG not increase the probability of CKD [53,55,59]; in the remaining studies, it was at least slightly increased. The latest research shows that prediabetes, defined through IGT or HbA1c [57,59,60], is a strong independent predicator for CKD.

A number of studies suggest that, in people with no DM, increased glycemia is related to an increased risk of albuminuria, which is believed to be the earliest marker for DKD. Already, in the NHANES, it was proved that the prevalence of microalbuminuria, and then macroalbuminuria, increased proportionally to worsening of glycemia—that is, from normal glycemia (6% microalbuminuria and 0.6% macroalbuminuria), to IFG (10% and 1.1%), undiagnosed diabetes (29% and 3.3%) and diagnosed diabetes (29% and 7.7%) [46]. Results of a prospective cohort study RENIS-T6 suggest that prediabetes plays an independent role in the development of glomerular hyperfiltration and albuminuria [61]. Discoveries of Melsom et al. indicate that there is a common pathophysiological background which leads to hyperfiltration and albuminuria in prediabetes and early stages of diabetes [62]. However, in the Chronic Renal Insufficiency Cohort (CRIC) study, in people with CKD, prediabetes was not related to an increased risk of eGFR decrease, yet it was related to an increased risk of proteinuria development and increased prevalence of cardiovascular diseases [63].

The range of glucose levels below the DM diagnostic threshold which increase the risk of albuminuria is not clear. Korean researchers who performed a cross-sectional study (n = 5202) divided participants on the basis of their FPG into 5 groups: <5.0, 5.0–5.5, 5.6–6.0, 6.1–6.9, and ≥7.0 mmol/L. In these groups, indicators of albuminuria were, respectively: 4.1%, 6.0%, 7.6%, 12.3%, and 23.4%, which shows an increase of the prevalence of albuminuria not only between normal FPG and IFG but also within the range of IFG [64].

Nevertheless, whether prediabetes can be seen as a cause of the development of CKD and albuminuria still remains unclear because other factors, e.g., hypertension, can also exert their impact adding to the effect of prediabetes [65,66].

#### 2.2.3. Diabetic Neuropathy

Diabetic neuropathy (DN) was considered to be a late complication of DM, yet there is more and more evidence showing its association with prediabetes. DN is the most frequently found complication of newly diagnosed DM [35]. In 18–25% of patients, there are changes in nerve function or their electromyograms results indicate that DN developed in the prediabetic stage. Prediabetes occurs in 25–62% of people with idiopathic peripheral neuropathy. Among people with prediabetes, 11–25% have peripheral neuropathy and 13–21% suffer from neuropathic pain [67]. In the MONICA/KORA study, it was shown that peripheral polyneuropathy appeared in 28% of people with DM, 13% of people with IGT, 11.3% of people with IFG, and 7.4% of people with no glucose metabolism disorders. In the same cohort, neuropathic pain was diagnosed in 13.3% of patients with DM, 8.7% of people with IGT, 4.2% of people with IFG, and 1.2% of people with no glucose metabolism disorders [68].

Additionally, there is more and more evidence indicating that, among people with IGT, the prevalence of idiopathic polyneuropathy, painful sensory neuropathy [69,70,71], and small-fiber neuropathy [72,73] is higher, while the motor fibers are less often affected [67,71]. Non-invasive assessment of neurological disorders in people with IGT revealed a greater prevalence of hyperesthesia, as well as hypoesthesia, and a higher threshold of heat detection [74]. Neuropathy in people with IGT seems to assume a milder course than in the case of DM patients because it affects mainly small not large nerve fibers [71]. Analogically to earlier data, there is evidence that the prevalence of DN increases proportionally to the gradient of glycemia—from people with normal glycemia, to people with IFG and IGT, to people with diabetes [75]. These findings suggest that small non-myelinated nerve fibers that convey pain and temperature, as well as regulating autonomic functions, are affected in prediabetes before DM develops fully.

A number of studies have concerned the association between autonomic disfunction and prediabetes [76,77,78,79,80,81,82,83,84,85,86,87,88,89,90,91] (Table 3). A great diversity of results was observed in these studies when it comes to the discussed glucose metabolism disorders, the size of the groups undergoing research, and cardiovascular autonomic neuropathy (CAN) testing methods. Only in four out of 16 studies were both IFG and IGT considered [81,83,87,89], and one study encompassed IFG + IGT [87]. In one study, IFG was defined in line with guidelines of the American Diabetes Association (ADA), from 1997—FPG 110–125 mg/dL (6.1–6.9 mmol/L) [79], while, in six studies, the IFG definition according to the 2003 ADA guidelines was followed—FPG 100–125 mg/dL (5.6–6.9 mmol/L) [80,81,82,83,87,89]. Nine studies followed IGT defined according to the WHO 1999 guidelines with 2-h post-load glucose levels between 140 and 199 mg/dL (7.8–11.0 mmol/L) in OGTT [78,81,84,85,86,87,88,89]. One study took into consideration an IFG-IGT group (defined according to ADA 2003 and WHO 1999 guidelines) [87], while two studies did not differentiate between these prediabetic states [90,91]. Seven studies included a small group (below 200 participants) [76,77,81,84,86,88,90], and six studies encompassed more than 1000 participants [76,79,80,82,83,91]. The size of groups undergoing research ranged from 80 to 9940. CAN (cardiovascular autonomic neuropathy) assessment tests were diversified: from standardized cardiovascular autonomic reflex tests (CARTs) to heart rate variability (HRV) measurements, but standard procedures and referential values related to the age of participants were not always applied [85,86]. Most of the analyzed studies did not provide the percentage of incorrect tests. In three studies, no differences were found between people with prediabetes and participants with NGT [76,77,84], while nine studies provided limited evidence to confirm lowered HRV results. In studies including both IFG and IGT, a tendency was observed that autonomic indicators in IGT were impaired more severely than in IFG [83,89], or, in the IGT + IFG group, in comparison with isolated IFG and IGT [87,92].

## 3. Future Prospects

Prediabetes is a heterogeneous nosological unit that may require the development of new diagnostic criteria. Recent studies suggest that HbA1_c_ should be included in the permanent diagnostic element due to its high specificity and provide modest improvements in risk discrimination for clinical complications [93]. The 1-h PG during the 75-g OGTT appears to be a useful early biomarker of dysglycemia, which is an indicator of prediabetes [94].

The research into new techniques for the diagnosis of microvascular complications in diabetes is still ongoing. Recent studies suggest that the expression of biomolecules, including microRNAs, proteins and metabolites, are specifically altered during the progression of prediabetes and T2DM and their complications. Their omnipresence in body fluids and the proven relationship of changes in their concentrations at the onset of the disease, during the progression and under treatment of carbohydrate metabolism disorders, make biomolecules a potential prognostic, diagnostic and progressive biomarker [95]. Sidorkiewicz et al. performed a baseline comparison of serum-circulating miRNAs in prediabetic individuals, distinguishing between those who later progressed to T2DM and those who did not [96]. Abnormal expression of long noncoding RNA (lncRNA) and mRNA in people with prediabetes has been shown, and it has been proven that lncRNAs are involved in the entire biological process of prediabetes [97,98,99].

Markers are investigated that may help in the diagnosis of complications in prediabetes in the future [100]. There are attempts to evaluate specific particles that can be used for the diagnosis of DKD [101,102] and DN [103]. Researchers proved that diabetic eye disease is not only DR but also damage to the front surface of the eye [41,104] and the formation of specific genetic biomarkers [105].

In order to inhibit the progression of glucose metabolism disorders, the emergence of DM, and microvascular complications, intensive therapeutic management based on behavioral and pharmacological interventions should be implemented [106]. In a study that compared the incidence of microvascular complications in subjects treated with non-pharmacological therapy versus subjects treated with metformin and/or linagliptin, no statistical difference was found [107]. Currently, clinicaltrials.gov has no data on studies assessing the effect of medicinal products on the incidence of microvascular complications in prediabetic states.

In addition to the potential effects of available antihyperglycemic drugs [108,109], it should be remembered that the hyperglycemia present in prediabetes can upregulate markers of chronic inflammation and contribute to the increased production of reactive oxygen species (ROS) that ultimately causes vascular dysfunction. Oxidative stress and inflammation in prediabetic states may become useful therapeutic points in the future, and targeted immunotherapies may prevent the progression of prediabetes to T2DM and the progression of complications [110].

## 4. Conclusions and Clinical Implications

The prevalence of prediabetes as a civilizational disease has been increasing, which is why it is necessary to establish a new set of diagnostic and therapeutic procedures. The current data indicate that there are microvascular complications of diabetes in people with impaired glucose metabolism in whom glycemia exceeds optimal values but does not yet match diagnostic criteria for DM. The kind and prevalence of diagnosed disorders differ depending on the type of prediabetic state, which may be affected by differences in pathophysiological mechanisms of their development. The proper diagnosis of prediabetic state is therefore crucial and should involve assessment of microvascular complications prevalence; thus, it should be based on applying new, more sensitive and specialized diagnostic tools. Due to the constantly growing number of people with prediabetic state, in order to inhibit the progression of glucose metabolism disorders, the development of DM, and microvascular complications, intensive therapeutic management based on behavioral and pharmacological interventions tailored to the patient’s individual needs should be implemented. Data on cardiovascular risk and all-cause mortality in people with microvascular complications are limited, but recent data indicate that pre-diabetes combined with prehypertension, but not prediabetes alone, in subjects without cardiovascular diseases might elevate the risk of all-cause mortality [111]. It is justified to continue research in this field in order to discover glucose thresholds which induce the development of microvascular complications of DM and to verify the currently applied criteria for diagnosing prediabetes and DM.

## Figures and Tables

**Table 1:** History of diagnostic criteria for prediabetes and diabetes mellitus (DM).

		WHO				ADA			CDA
		1965 [6]	1980 [7]	1985 [8]	1999, 2006 [9]	1997 [10]	2003 [11]	2010 [12]	2018 [13]
Prediabetes	*At least 1 of the conditions:*								
	FPG (mg/dL)		120–139	<140	110–125 ^a^	110–125 ^a^	100–125 ^a^	100–125 ^a^	110–125 ^a^
	2-h PG OGTT (mg/dl)	110–129 ^#^	140–200	140–200	140–199 ^b^	140–199 ^b^	140–199 ^b^	140–199 ^b^	140–199 ^b^
	HbA1_c_ (%)							5.7–6.4%	6.0–6.4%
Diabetes	*At least 1 of the conditions:*								
	FPG (mg/dL)	≥130 *	≥140	≥140	≥126	≥126	≥126	≥126	≥126
	2-h PG OGTT (mg/dl)	≥130	≥200	≥200	≥200	≥200	≥200	≥200	≥200
	HbA1_c_ (%)							≥6.5%	≥6.5%

* + glucosuria; ^#^ 50 g lub 100 g glucose in OGTT; ^a^ IFG, impaired fasting glucose; ^b^ IGT, impaired glucose tolerance; WHO, World Health Organization; ADA, American Diabetes Association; CDA, Canadian Diabetes Association; FPG, fasting plasma glucose; 2-h PG, 2-h plasma glucose; OGTT, oral glucose tolerance test; HbA1_c_, glycated hemoglobin.

**Table 2:** Characteristics of cohort studies on the association between prediabetes and risk of chronic kidney disease.

Study and Year	Population	Number (n)	Ethnicity	Age (Years)	Criteria	Outcome Definition	Follow-Up (Years)	Number of Events	Effect Estimate: OR or RR (95% CI)	DKD Correlates
Fox et al. (2005) [47]	All	2398	Mainly white	28–62	IFG: FPG 100–125 mg/dL; IGT: 2-h PG 140–199 mg/dL	CKD (eGFR <60 mL/min/1.73 m^2^)—MDRD study equation	7	167		age, sex, baseline eGFR, SBP, AH treatment, smoking, BMI, TC, HDL, and prevalent MI or congestive HF
	NGT	1502								
	IFG or IGT	704							0.98 (0.67–1.45)	
	n-DM	82							1.71 (0.83–3.55)	
	k-DM	110							1.93 (1.06–3.49)	
Kurella et al. (2005) [48]	All	10,096	Mainly white, Black (20%)	45–64	IFG: FPG ≥ 110 mg/dl	CKD (eGFR <60 mL/min/1.73 m^2^)—MDRD study equation	9	691	1.11 (0.87–1.40)	age, gender, race
	IFG	691								
Tozawa et al. (2007) [49]	All	6371	Asian/Japanese	19-84	IFG: FPG ≥ 110 mg/dl	CKD defined as dipstick positive proteinuria (≥1+) or eGFR <60 mL/min/1.73 m^2^ using the MDRD study equation	5	369	1.22 (0.89–1.67)	age, sex, current, cigarette smoking, and alcohol drinking habits
Lucove et al. (2008) [50]	All	2420	American Indians	45–74	IFG: FPG ≥ 110 mg/dl	CKD defined as ACR ≥30 mg/g or eGFR <60 ml/min/1.73 m^2^ using the MDRD study equation	9	388	1.4 (1.1–1.8)	age, sex, study center, and cigarette smoking
	IFG	605								
Rashidi et al. (2007) [51]	All	4607	Asian/Iranian	>18	IFG: FPG ≥ 110 mg/dL	CKD (eGFR <60 mL/min/1.73 m^2^)—Cockroft–Gault study equation	3	111	1.02 (1.00–1.04)	Not provided
Ryu et al. (2009) [52]	All	10685	Asian/Korean	30–59	IFG: FPG ≥ 110 mg/dl	CKD (eGFR <60 mL/min/1.73 m^2^)—MDRD study equation	3.8	291	1.61 (0.95–2.73)	age, baseline eGFR, γ-glutamyl transpeptidase level, uric acid level, obesity, increased TG, low HDL, increased BP
Sun et al. (2010) [53]	All	118,924	Asian/Chinese	20–74	IFG: FPG ≥ 100 mg/dL	CKD defined as dipstick positive proteinuria (≥ 1+) or eGFR <60 mL/min/1.73 m^2^ using the MDRD study equation	3.7	12648	0.97 (0.93–1.01)	age, sex, check-up centers and current smoking
	IFG	28,827								
Watanabe et al. (2012) [54]	All	27,359	Asian/Japanese	≥20	IFG: FPG ≥ 110 mg/dL	CKD (eGFR < 60 mL/min/1.73 m^2^)—MDRD study equation	5.8	513	1.84 (1.42–2.38)	age and sex
Schöttker et al. (2013) [55]	All	3538	Caucasian	50–74	IFG: FPG ≥ 110 mg/dL; eHbA_1c_: 39−47 mmol/mol	CKD (eGFR < 60 mL/min/1.73 m^2^)—CKD EPI study equation	8		0.97 (0.75–1.25)	age, sex, baseline eGFR, BMI, SBP (continuous), TC, use of antihypertensive drugs, use of statins, smoking status and history of self-reported cardiovascular disease (MI or stroke)
	normal FPG	2076						364	0.97 (0.75–1.25)	
	IFG	516						87		
	normal HbA_1c_	2014						321	1.03 (0.86–1.23)	
	eHbA1c	1034						213		
	normal FPG and HbA_1c_	1471						235	1.06 (0.71–1.32)	
	both IFG and eHbA_1c_	258						55		
Jadhakhan et al. (2018) [56]	All	40,092	Mainly white and Asian	18–40	IFG: FPG ≥ 110 mg/dL; IGT: 2-h gc ≥ 140 mg/dL ≤ 199 mg/dL; eHbA_1c_: 42–47 mmol/mol	CKD (eGFR < 60 ml/min/1.73 m^2^)—CKD EPI study equation	2		2.6 (2.0–3.4)	age, sex, ethnic group, deprivation quintile, BMI, CVD, HF, AF, AH, and steroid use
	NGT	29,531						126		
	IFG and IGT	10,561						182		
Koshi et al. (2018) [57]	All	25,109	Asian/Japanese	≥18	Prediabetes 1: FPG 100–125 mg/dl and/or eHbA_1c_ 39−47 mmol/mol; Prediabetes 2: FPG 110–125 mg/dL and/or eHbA1c: 42–47 mmol/mol	eGFR <60 mL/min/1.73 m2 and/or dipstick positive proteinuria—IDMS-MDRD study equation	5.3	2483		sex, age, insulin sensitivity, SBP, baseline eGFR, serum alanine aminotransferase level
	Prediabetes_ADA_	10,367							1.21 (1.12–1.32)	
	Prediabetes_WHO_	2561							1.31 (1.16–1.48)	
Vieira et al. (2019) [58]	All	9361	Main white (55%), Black (32%), Hispanic (10%)	≥50	IFG: FPG ≥ 100 mg/dL	Worsening kidney function ^a^ or albuminuria	3.3	193	1.02 (0.75–1.37)	age, sex, race, smoking status, SBP, prior CVD, BMI, statin use, aspirin use, and trial treatment arm
	NGT	5424						114		
	IFG	3897						79		
Kim et al. (2020) [59]	All	7728	Asian/Korean	≥18	IFG: 110–125 mg/dL; IGT: 2-h gc ≥ 140 mg/dL ≤ 199 mg/dL; eHbA_1c_: 39–47 mmol/mol	CKD (eGFR < 60 mL/min/1.73 m^2^)—CKD EPI study equation	8.7	871		age >65, sex, AH, obesity, regular activity, baseline eGFR, and metabolic syndrome
	IFG	652						84	0.92 (0.72–1.17)	
	IGT	1882						297	1.14 (1.18–1.31)	
	eHbA1c	2461						425	1.39 (1.21–1.60)	
Li et al. (2020) [60]	All	6446	Asian/Chinese	40–84	IFG: FPG 100–125 mg/dL; IGT: 2-h gc ≥ 140 mg/dL ≤ 199 mg/dL; eHbA1c: 39−47 mmol/mo	CKD (eGFR < 60 mL/min/1.73 m^2^)—CKD EPI study equation	3	88		age, sex, BMI, TC, TG, HDL, LDL, SBP, DBP, baseline eGFR, smoking and drinking status
	NGT	1836						10		
	Prediabetes	4610						78	2.45 (1.26–4.81)	
	IFG	1954						38	2.51 (1.21–5.20)	
	IGT	1696						36	2.39 (1.14–5.03)	
	eHBA1c	3848						65	2.60 (1.31-5.16)	

^a^ In participants without CKD at baseline, worsening kidney function was defined by a decrease in the eGFR of 30% or more to a value of <60 mL/min/1.73 m^2^. In participants with CKD at baseline, worsening kidney function was defined by a decrease in the eGFR of 50% or more or the development of ESRD requiring long-term dialysis or kidney transplantation; CKD, chronic kidney disease; eGFR, estimated glomerular filtration rate; ESRD, end-stage renal disease; MDRD, Modification of Diet in Renal Disease; CKD-EPI, Chronic Kidney Disease Epidemiology Collaboration; IDMS-MDRD, isotope dilution mass spectrometry traceable 4-variable Modification of Diet in Renal Disease; ACR, albumin-to-creatinine ratio; NGT, normal glucose tolerance; PG, plasma glucose; FPG, fasting plasma glucose; IGT, impaired glucose tolerance; IFG; impaired fasting glucose; k-DM, known diabetes mellitus; n-DM, newly detected diabetes mellitus; BMI, body mass index; HOMA-I, homeostatic model assessment index; SBP, systolic blood pressure; DBP, diastolic blood pressure; BP, blood pressure; AH, arterial hypertension; MI, myocardial infarction; HF, heart failure; CVD, cardiovascular disease; AF, atrial fibrillation; HR, heart rate; HbA_1c_, glycated hemoglobin; eHbA1c, elevated glycated hemoglobin; TC, total cholesterol; HDL, high-density lipoprotein; LDL, low-density lipoprotein, TG, triglycerides; ADA, American Diabetes Association; WHO, World Health Organization.

**Table 3:** Studies evaluating the presence of cardiovascular autonomic neuropathy (CAN) in pre-diabetes.

Study and Year	Population	Number (n)	Criteria	Test (s) Used	Prevalence	Differences vs. NGT	CAN Correlates
Annuzzi et al. (1983) [76]	All	186	EASD 1979	DB	Not provided	No difference	Age, BMI
	NGT	124					
	IGT	62					
Fujimoto et al. (1987) [77]	All	151	WHO 1980	DB	Not provided	No difference	Fasting glucose concentration
	NGT	79					
	IGT	72					
	k-DM	78					
Gerritsen et al. (2000) [78]	All	631	WHO 1985	Short-term HRV, BRS, DB, LS, OH	Not provided	↓SDNN	Age, antihypertensive drugs, presence of diabetes
	NGT	288					
	IGT	169					
	n-DM	95					
	k-DM2	79					
Singh et al. (2000) [79]	All	1919	WHO 1985	Short-term HRV	Not provided	↓SDNN, HF, and LF in IFG and DM	Fasting glucose
	NFG	1779					
	IFG	56					
	DM	84					
Schroeder et al. (2005) [80]	All	9940	ADA 2003	Short-term HRV	Not provided	↓RR interval and rMSSD in IFG at baseline	Fasting glucose and HRV (weak association at baseline) in nondiabetic subjects
	NFG	5410					
	IFG	3561				↓SDNN, rMSSD, and R-R interval in DM at baseline	
	DM	969					
Perciaccante et al. (2006) [81]	All	100	ADA 2003	24-h HRV	Not provided	↓SDNN, low TP, and ↑LFnu in DM, IFG, and IGT	HOMA-I
	NGT	20					
	IFG	20				↑LFnu in IFG and IGT than in DM and NGT	
	IGT	20					
	DM2	20				↑LFnu in NGT than IFG	
	NGT without insulin resistance	20				↓HF in NGR and IGT than IFG and DM	
Stein et al. (2007) [82]	All	1259	ADA 2003	24-h HRV	Not provided	↓RR interval, SDNN and TP in IFG-2 and DM	Fasting glucose, metabolic syndrome components
	NFG	536					
	IFG-1	363					
	IFG-2	182					
	DM	178					
Wu et al. (2007) [83]	All	1440	ADA 2003	Short-term HRV, DB, LS	Not provided	↓DB in DM than IGT	Not provided
	NGT	983				↓SDNN and DB in IFG	
	i-IFG	163				↓SDNN, LS, HF power and DB, but ↑LF:HF ratio in DM and IGF	
	IGT	188					
	DM	106					
Isak et al. (2008) [84]	All	50	ADA 2003	DB, LS, VM, OH, Handgrip, Sudomotor function	Not provided	No differences apart from in sympathetic skin response	Not provided
	NGT	25					
	IGT	25					
Laitinen et al. (2011) [85]	All	268	ADA 2003	DB, OH	25% abnormal DB	Not provided (no control group)	Age, weight, BMI, waist, SBP, triglycerides, sex (male) in DB
	IGT	268			6% abnormal OH		SBP in OH
Putz et al. (2013) [86]	All	115	ADA 2003	DB, LS, VM, OH, Handgrip test, Triangle index	IGT: 57.5% one abnormal test	↓DB, Valsalva ratio, OH, handgrip test, and triangle index	Not provided
	NFG	40					
	IGT	75					
Ziegler et al. (2015) [87]	All	1332	ADA 2003	4 Out of 120 short-term HRV indices		4 in i-IFG and 6 in IFG-IGT and n-DM HRV measures more frequently abnormal	HR, BMI, hypertension, smoking, creatinine, sex, age, HbA1_c_, physical activity, drugs suppressing HRV as predictors of diminished HRV
	NGT	565			4.5%		
	i-IFG	336			8.1%		
	i-IGT	72			5.9%		
	IFG-IGT	151			11.4%		
	n-DM	78			11.7%		
	k-DM	130			17.5%		
Tiftikcioglu et al. (2016) [88]	All	80	ADA 2003	short-term HRV, Sudomotor function	Not provided	↓SDNN, CV, TP, LF, LF: HF in IGT and DM	Not provided
	NGT	30					
	IGT	25					
	DM	25					
Dimova et al. (2016) [89]	All	478	WHO 2006	short-term HRV indices		↓Sympathetic and parasympathetic tone in IFG, iIGT and DM	Age, QTc-i, waist for sympathetic and parasympathetic indices
	NGT	130			12.3%		
	preDM	227			19.8%		
	IFG	125			13.2%		
	iIGT	102			20.6%		DBP, 2-h BG for sympathetic indices
	NDT1DM	121			32.2%		
Dimova et al. (2019) [90]	All	87	WHO 2006	short-term HRV indices, DB, VM, LS		↓parasympathetic activity at rest in n-DM	Age, hypertension, waist, BMI, visceral fat, total body fat
	NGT	35			5.7%		
	Prediabetes	35			8.6%	↑HR at rest in n-DM	
	n-DM	17			23.5%		
Coopmans et al. (2020) [91]	All	2107	WHO 2006	24 h HRV	Not provided	↓time and frequency domain HRV in prediabetes and DM	Age, female sex, BMI, HDL-cholesterol, alcohol use, smoking behavior, HbA1_c_, FPG, 2-h BG
	NGT	1226					
	Prediabetes	331					
	DM	550					

CAN, cardiovascular autonomic neuropathy; NGT, normal glucose tolerance; FPG, fasting plasma glucose; IGT, impaired glucose tolerance; i-IGT, isolated impaired glucose tolerance IFG; impaired fasting glucose; IFG-1, 5.6–6.0 mmol/L; IFG-2, 6.1–6.9 mmol/L; i-IFG, isolated impaired fasting glucose; k-DM, known diabetes mellitus; n-DM, newly detected diabetes mellitus; T2DM, type 2 diabetes mellitus; DB, deep breathing; HRV, heart rate variability; BRS, baroreflex sensitivity; LS, lying to standing; OH, orthostatic hypotension; VM, Valsalva maneuver; SDNN, standard deviation of NN intervals; HF, high-frequency spectral component of heart rate variability; LF, low-frequency spectral component of heart rate variability; RR, coefficient of variation; rMSSD, root means successive square difference; TP, total power of heart rate variability; nu, normalized units; BMI, body mass index; HOMA-I, homeostatic model assessment index; QTc-i, corrected QT interval; SBP, systolic blood pressure; DBP, diastolic blood pressure; BG, blood glucose; BP, blood pressure; HR, heart rate; HbA1C, glycated hemoglobin.

## References

[1] (1999). Definition, Diagnosis and Classification of Diabetes Mellitus and Its Complications: Report of a WHO Consultation. Part 1, Diagnosis and Classification of Diabetes Mellitus.

[2] International Diabetes Federation (2019). IDF Diabetes Atlas.

[3] Brannick B., Wynn A., Dagogo-Jack S. (2016). Prediabetes as a toxic environment for the initiation of microvascular and macrovascular complications. Exp. Biol. Med..

[4] Hostalek U. (2019). Global epidemiology of prediabetes—Present and future perspectives. Clin. Diabetes Endocrinol..

[5] Štěpánek L., Horakova D., Nakládalová M., Cibickova L., Karasek D., Zadrazil J. (2018). Significance of prediabetes as a nosological entity. Biomed. Pap..

[6] WHO (1965). Diabetes mellitus. Report of a WHO expert committee. World Health Organ. Tech. Rep. Ser..

[7] WHO (1980). WHO Expert Committee on Diabetes Mellitus: Second report. World Health Organ. Tech. Rep. Ser..

[8] WHO Study Group on Diabetes Mellitus, World Health Organization (1985). Diabetes Mellitus: Report of a WHO Study Group.

[9] World Health Organization, International Diabetes Federation (2006). Definition and Diagnosis of Diabetes Mellitus and Intermediate Hyperglycaemia: Report of a WHO/IDF Consultation.

[10] American Diabetes Association (1997). Report of the Expert Committee on the Diagnosis and Classification of Diabetes Mellitus. Diabetes Care.

[11] American Diabetes Association (2003). Report of the Expert Committee on the Diagnosis and Classification of Diabetes Mellitus. Diabetes Care.

[12] (2010). Standards of Medical Care in Diabetes—2010. Diabetes Care.

[13] Punthakee Z., Goldenberg R., Katz P. (2018). Definition, classification and diagnosis of diabetes, prediabetes and metabolic syndrome. Can. J. Diabetes.

[14] Bullard K.M., Saydah S.H., Imperatore G., Cowie C.C., Gregg E.W., Geiss L.S., Cheng Y.J., Rolka D.B., Williams D.E., Caspersen C.J. (2013). Secular changes in U.S. prediabetes prevalence defined by hemoglobin a1c and fasting plasma glucose: National health and nutrition examination Surveys, 1999–2010. Diabetes Care.

[15] Centers for Disease Control and Prevention (2020). National Diabetes Statistics Report.

[16] Fonville S., Zandbergen A.A., Koudstaal P.J., Hertog H.M.D. (2014). Prediabetes in patients with stroke or transient ischemic attack: Prevalence, risk and clinical management. Cerebrovasc. Dis..

[17] Tabák Á.G., Herder C., Rathmann W., Brunner E.J., Kivimäki M. (2012). Prediabetes: A high-risk state for diabetes development. Lancet.

[18] American Diabetes Association (2019). Microvascular complications and foot care: Standards of medical care in diabetes−2020. Diabetes Care.

[19] American Diabetes Association (2019). Cardiovascular disease and risk management: Standards of medical care in diabetes—2020. Diabetes Care.

[20] Abdul-Ghani M.A., Tripathy D., DeFronzo R.A. (2006). Contributions of -cell dysfunction and insulin resistance to the pathogenesis of impaired glucose tolerance and impaired fasting glucose. Diabetes Care.

[21] Danaei G., Finucane M.M., Lu Y., Singh G.M., Cowan M.J., Paciorek C.J., Lin J.K., Farzadfar F., Khang Y.H., Stevens G.A. (2011). National, regional, and global trends in fasting plasma glucose and diabetes prevalence since 1980: Systematic analysis of health examination surveys and epidemiological studies with 370 country-years and 2·7 million participants. Lancet.

[22] Ferrannini E., Gastaldelli A., Iozzo P. (2011). Pathophysiology of prediabetes. Med. Clin. N. Am..

[23] DeFronzo R.A. (2009). From the triumvirate to the ominous octet: A new paradigm for the treatment of type 2 diabetes mellitus. Diabetes.

[24] Tabák A.G., Jokela M., Akbaraly T.N., Brunner E.J., Kivimäki M., Witte D. (2009). Trajectories of glycaemia, insulin sensitivity, and insulin secretion before diagnosis of type 2 diabetes: An analysis from the Whitehall II study. Lancet.

[25] Sattar N., McConnachie A., Ford I., Gaw A., Cleland S.J., Forouhi N.G., McFarlane P., Shepherd J., Cobbe S., Packard C. (2007). Serial metabolic measurements and conversion to type 2 diabetes in the west of Scotland coronary prevention study: Specific elevations in alanine aminotransferase and triglycerides suggest hepatic fat accumulation as a potential contributing factor. Diabetes.

[26] Mason C.C., Hanson R.L., Knowler W.C. (2007). Progression to type 2 diabetes characterized by moderate then rapid glucose increases. Diabetes.

[27] Ferrannini E., Nannipieri M., Williams K., Gonzales C., Haffner S.M., Stern M.P. (2004). Mode of onset of type 2 diabetes from normal or impaired glucose tolerance. Diabetes.

[28] Gastaldelli A., Ferrannini E., Miyazaki Y., Matsuda M., DeFronzo R.A. (2004). Beta-cell dysfunction and glucose intolerance: Results from the San Antonio metabolism (SAM) study. Diabetologia.

[29] Weir G.C., Bonner-Weir S. (2004). Five stages of evolving beta-cell dysfunction during progression to diabetes. Diabetes.

[30] Kanat M., DeFronzo R.A., Abdul-Ghani M.A. (2015). Treatment of prediabetes. World J. Diabetes.

[31] DeFronzo R.A., Abdul-Ghani M.A. (2011). Preservation of β-cell function: The key to diabetes prevention. J. Clin. Endocrinol. Metab..

[32] Kanat M., Mari A., Norton L., Winnier D., DeFronzo R.A., Jenkinson C., Abdul-Ghani M.A. (2012). Distinct β-cell defects in impaired fasting glucose and impaired glucose tolerance. Diabetes.

[33] Abdul-Ghani M., DeFronzo R.A., Jayyousi A. (2016). Prediabetes and risk of diabetes and associated complications. Curr. Opin. Clin. Nutr. Metab. Care.

[34] Brownlee M. (2005). The pathobiology of diabetic complications: A unifying mechanism. Diabetes.

[35] Bansal N. (2015). Prediabetes diagnosis and treatment: A review. World J. Diabetes.

[36] Diabetes Prevention Program Research Group (2007). The prevalence of retinopathy in impaired glucose. Diabetic Med..

[37] Kawasaki R., Wang J.J., Wong T.Y., Kayama T., Yamashita H. (2008). Impaired glucose tolerance, but not impaired fasting glucose, is associated with retinopathy in Japanese population: The Funagata study. Diabetes Obes. Metab..

[38] Lamparter J., Raum P., Pfeiffer N., Peto T., Hoehn R., Elflein H., Wild P., Schulz A., Schneider A., Mirshahi A. (2014). Prevalence and associations of diabetic retinopathy in a large cohort of prediabetic subjects: The gutenberg health study. J. Diabetes Complicat..

[39] Wong T.Y., Liew G., Tapp R.J., Schmidt M.I., Wang J.J., Mitchell P., Klein R., Klein B.E.K., Zimmet P., Shaw J. (2008). Relation between fasting glucose and retinopathy for diagnosis of diabetes: Three population-based cross-sectional studies. Lancet.

[40] Tyrberg M., Melander A., Lövestam-Adrian M., Lindblad U., Lvestam-Adrian M. (2008). Retinopathy in subjects with impaired fasting glucose: The NANSY-Eye baseline report. Diabetes Obes. Metab..

[41] Azmi S., Ferdousi M., Petropoulos I.N., Ponirakis G., Alam U., Fadavi H., Asghar O., Marshall A., Atkinson A.J., Jones W.T. (2015). Corneal confocal microscopy identifies small-fiber neuropathy in subjects with impaired glucose tolerance who develop type 2 diabetes. Diabetes Care.

[42] Cheng Y.J., Gregg E.W., Geiss L.S., Imperatore G., Williams D.E., Zhang X., Albright A.L., Cowie C.C., Klein R., Saaddine J.B. (2009). Association of A1C and Fasting Plasma Glucose Levels With Diabetic Retinopathy Prevalence in the U.S. Population: Implications for diabetes diagnostic thresholds. Diabetes Care.

[43] Lee M., Hsu W., Lai C., Chen S., Liang C. (2019). The association between glycated albumin, glycohemoglobin, and glycated albumin to glycohemoglobin ratio in diabetic retinopathy of prediabetes. Kaohsiung J. Med. Sci..

[44] Nguyen T.T., Wang J.J., Wong T.Y. (2007). Retinal Vascular Changes in Pre-Diabetes and Prehypertension: New findings and their research and clinical implications. Diabetes Care.

[45] Zaleska-Żmijewska A., Piątkiewicz P., Śmigielska B., Sokołowska-Oracz A., Wawrzyniak Z., Romaniuk D., Szaflik J., Szaflik J.P. (2017). Retinal photoreceptors and microvascular changes in prediabetes measured with adaptive optics (rtx1™): A case-control study. J. Diabetes Res..

[46] Plantinga L.C., Crews D.C., Coresh J., Miller E.R., Saran R., Yee J., Hedgeman E., Pavkov M., Eberhardt M.S., Williams D.E. (2010). Prevalence of chronic kidney disease in US adults with undiagnosed diabetes or prediabetes. Clin. J. Am. Soc. Nephrol..

[47] Fox C.S., Larson M.G., Leip E.P., Meigs J.B., Wilson P.W., Levy D. (2005). Glycemic status and development of kidney disease: The framingham heart study. Diabetes Care.

[48] Kurella M., Lo J.C., Chertow G.M. (2005). Metabolic syndrome and the risk for chronic kidney disease among nondiabetic adults. J. Am. Soc. Nephrol..

[49] Tozawa M., Iseki C., Tokashiki K., Chinen S., Kohagura K., Kinjo K., Takishita S., Iseki K., Tozawa C.I.M. (2007). Metabolic syndrome and risk of developing chronic kidney disease in Japanese adults. Hypertens. Res..

[50] Lucove J., Vupputuri S., Heiss G., North K., Russell M. (2008). Metabolic syndrome and the development of CKD in American Indians: The strong heart study. Am. J. Kidney Dis..

[51] Rashidi A., Ghanbarian A., Azizi F. (2007). Are patients who have metabolic syndrome without diabetes at risk for developing chronic kidney disease? Evidence based on data from a large cohort screening population. Clin. J. Am. Soc. Nephrol..

[52] Ryu S., Chang Y., Woo H.Y., Lee K.B., Kim S.G., Kim D.I., Kim W.S., Suh B.S., Jeong C., Yoon K. (2009). Time-dependent association between metabolic syndrome and risk of CKD in Korean men without hypertension or diabetes. Am. J. Kidney Dis..

[53] Sun F., Tao Q., Zhan S. (2010). Metabolic syndrome and the development of chronic kidney disease among 118 924 non-diabetic Taiwanese in a retrospective cohort. Nephrology.

[54] Watanabe H., Obata H., Watanabe T., Sasaki S., Nagai K., Aizawa Y. (2010). Metabolic syndrome and risk of development of chronic kidney disease: The Niigata preventive medicine study. Diabetes Metab. Res. Rev..

[55] Schöttker B., Brenner H., Koenig W., Müller H., Rothenbacher D. (2013). Prognostic association of HbA1c and fasting plasma glucose with reduced kidney function in subjects with and without diabetes mellitus. Results from a population-based cohort study from Germany. Prev. Med..

[56] Jadhakhan F., Marshall T., Ryan R., Gill P. (2018). Risk of chronic kidney disease in young adults with impaired glucose tolerance/impaired fasting glucose: A retrospective cohort study using electronic primary care records. BMC Nephrol..

[57] Koshi T., Sagesaka H., Sato Y., Hirabayashi K., Koike H., Yamauchi K., Nishimura R., Noda M., Yamashita K., Aizawa T. (2018). Elevated haemoglobin A1c but not fasting plasma glucose conveys risk of chronic kidney disease in non-diabetic individuals. Diabetes Res. Clin. Pract..

[58] Vieira M.B., Neves J.S., Leitão L., Baptista R.B., Magriço R., Dias C.V., Oliveira A., Carvalho D., Mc Causland F.R. (2019). Impaired fasting glucose and chronic kidney disease, albuminuria, or worsening kidney function: A secondary analysis of the SPRINT. J. Clin. Endocrinol. Metab..

[59] Kim G.S., Oh H.H., Kim S.H., Kim B.O., Byun Y.S. (2019). Association between prediabetes (defined by HbA1C, fasting plasma glucose, and impaired glucose tolerance) and the development of chronic kidney disease: A 9-year prospective cohort study. BMC Nephrol..

[60] Li W., Wang A., Jiang J., Liu G., Wang M., Li D., Wen J., Mu Y., Du X., Gaisano H. (2020). Risk of chronic kidney disease defined by decreased estimated glomerular filtration rate in individuals with different prediabetic phenotypes: Results from a prospective cohort study in China. BMJ Open Diabetes Res. Care.

[61] Melsom T., Schei J., Stefansson V.T.N., Solbu M.D., Jenssen T.G., Mathisen U.D., Wilsgaard T., Eriksen B.O. (2016). Prediabetes and risk of glomerular hyperfiltration and albuminuria in the general nondiabetic population: A prospective cohort study. Am. J. Kidney Dis..

[62] De Nicola L., Conte G., Minutolo R. (2016). Prediabetes as a precursor to diabetic kidney disease. Am. J. Kidney Dis..

[63] Neves J.S., Correa S., Baptista R.B., Vieira M.B., Waikar S.S., Mc Causland F.R. (2020). Association of prediabetes with CKD progression and adverse cardiovascular outcomes: An analysis of the CRIC Study. J. Clin. Endocrinol. Metab..

[64] Won J.C., Hong J.W., Kim J.M., Kim T.N., Noh J., Ko K.S., Rhee B.D., Kim D.-J. (2015). Increased prevalence of albuminuria in individuals with higher range of impaired fasting glucose: The 2011 Korea National Health and Nutrition Examination Survey. J. Diabetes Complicat..

[65] Thomas G., Sehgal A.R., Kashyap S.R., Srinivas T.R., Kirwan J.P., Navaneethan S.D. (2011). Metabolic syndrome and kidney disease: A systematic review and meta-analysis. Clin. J. Am. Soc. Nephrol..

[66] Curhan G.C. (2010). Prediabetes, prehypertension … is it time for pre-CKD?. Clin. J. Am. Soc. Nephrol..

[67] Papanas N., Vinik A.I., Ziegler D. (2011). Neuropathy in prediabetes: Does the clock start ticking early?. Nat. Rev. Endocrinol..

[68] Ziegler D., Rathmann W., Dickhaus T., Meisinger C., Mielck A. (2009). Neuropathic pain in diabetes, prediabetes and normal glucose tolerance: The MONICA/KORA Augsburg surveys S2 and S3. Pain Med..

[69] Snyder C.R.H., Smith B.E., Ross M.A., Hernandez J., Bosch E.P. (2006). Value of the oral glucose tolerance test in the evaluation of chronic idiopathic axonal polyneuropathy. Arch. Neurol..

[70] Nebuchennykh M., Lseth S., Jorde R., Mellgren S.I., Løseth S. (2008). Idiopathic polyneuropathy and impaired glucose metabolism in a Norwegian patient series. Eur. J. Neurol..

[71] Sumner C., Sheth S., Griffin J., Cornblath D., Polydefkis M. (2003). The spectrum of neuropathy in diabetes and impaired glucose tolerance. Neurology.

[72] Singleton J.R., Smith A.G., Bromberg M.B. (2001). Increased prevalence of impaired glucose tolerance in patients with painful sensory neuropathy. Diabetes Care.

[73] Smith A.G., Ramachandran P., Tripp S., Singleton J.R. (2001). Epidermal nerve innervation in impaired glucose tolerance and diabetes-associated neuropathy. Neurology.

[74] Putz Z., Tabák Á.G., Tóth N., Istenes I., Németh N., Gandhi R.A., Hermányi Z., Keresztes K., Jermendy G., Tesfaye S. (2009). Noninvasive evaluation of neural impairment in subjects with impaired glucose tolerance. Diabetes Care.

[75] Ziegler D., Papanas N., Vinik A.I., Shaw J.E. (2014). Epidemiology of polyneuropathy in diabetes and prediabetes. Handbook of Clinical Neurology.

[76] Annuzzi G., Rivellese A., Vaccaro O., Ferrante M.R., Riccardi G., Mancini M. (1984). The relationship between blood glucose concentration and beat-to-beat variation in asymptomatic subjects. Acta Diabetol. Lat..

[77] Fujimoto W.Y., Leonetti D.L., Kinyoun J.L., Shuman W.P., Stolov W.C., Wahl P.W. (1987). Prevalence of complications among second-generation Japanese-American men with diabetes, impaired glucose tolerance, or normal glucose tolerance. Diabetes.

[78] Gerritsen J., Dekker J.M., TenVoorde B.J., Bertelsmann F.W., Kostense P.J., Stehouwer C.D.A., Heine R.J., Nijpels G., Heethaar R.M., Bouter L.M. (2000). Glucose tolerance and other determinants of cardiovascular autonomic function: The Hoorn Study. Diabetologia.

[79] Singh J.P., Larson M.G., O’Donnell C.J., Wilson P.F., Tsuji H., Lloyd-Jones D.M., Levy D. (2000). Association of hyperglycemia with reduced heart rate variability (The Framingham Heart Study). Am. J. Cardiol..

[80] Schroeder E.B., Chambless L.E., Liao D., Prineas R.J., Evans G.W., Rosamond W.D., Heiss G. (2005). Diabetes, glucose, insulin, and heart rate variability: The atherosclerosis risk in communities (ARIC) study. Diabetes Care.

[81] Perciaccante A., Fiorentini A., Paris A., Serra P., Tubani L. (2006). Circadian rhythm of the autonomic nervous system in insulin resistant subjects with normoglycemia, impaired fasting glycemia, impaired glucose tolerance, type 2 diabetes mellitus. BMC Cardiovasc. Disord..

[82] Stein P.K., Barzilay J.I., Domitrovich P.P., Chaves P.M., Gottdiener J.S., Heckbert S.R., Kronmal R.A. (2007). The relationship of heart rate and heart rate variability to non-diabetic fasting glucose levels and the metabolic syndrome: The cardiovascular health study. Diabet. Med..

[83] Wu J.S., Lin T.S., Huang Y.H., Lu F.H., Yang Y.C., Chen J.J., Chang C.J. (2007). Epidemiological evidence of altered cardiac autonomic function in subjects with impaired glucose tolerance but not isolated impaired fasting glucose. J. Clin. Endocrinol. Metab..

[84] Isak B., Tanrıdağ T., Yitmen I., Us O., Oflazoğlu B. (2008). Evaluation of peripheral and autonomic neuropathy among patients with newly diagnosed impaired glucose tolerance. Diabetes Metab. Res. Rev..

[85] Laitinen T., Lindström J., Eriksson J.G., Ilanne-Parikka P., Aunola S., Keinänen-Kiukaanniemi S., Tuomilehto J., Uusitupa M. (2011). Cardiovascular autonomic dysfunction is associated with central obesity in persons with impaired glucose tolerance. Diabet. Med..

[86] Putz Z., Nemeth N., Istenes I., Martos T., Gandhi R.A., Körei A.E., Hermanyi Z., Szathmári M., Jermendy G., Tesfaye S. (2013). Autonomic dysfunction and circadian blood pressure variations in people with impaired glucose tolerance. Diabet. Med..

[87] Ziegler D., Voss A., Rathmann W., Strom A., Perz S., Roden M., Peters A., Meisinger C. (2015). Increased prevalence of cardiac autonomic dysfunction at different degrees of glucose intolerance in the general population: The KORA S4 survey. Diabetologia.

[88] Tiftikçioğlu B.I., Bilgin S., Duksal T., Kose S., Zorlu Y. (2016). Autonomic neuropathy and endothelial dysfunction in patients with impaired glucose tolerance or type 2 diabetes mellitus. Medicine.

[89] Dimova R., Tankova T., Guergueltcheva V., Tournev I., Chakarova N., Grozeva G., Dakovska L. (2017). Risk factors for autonomic and somatic nerve dysfunction in different stages of glucose tolerance. J. Diabetes Complicat..

[90] Dimova R., Tankova T., Kirilov G., Chakarova N., Grozeva G., Dakovska L. (2020). Endothelial and autonomic dysfunction at early stages of glucose intolerance and in metabolic syndrome. Horm. Metab. Res..

[91] Coopmans C., Zhou T.L., Henry R.M., Heijman J., Schaper N.C., Koster A., Schram M.T., Van Der Kallen C.J., Wesselius A., Engelsman R.J.D. (2020). Both prediabetes and type 2 diabetes are associated with lower heart rate variability: The maastricht study. Diabetes Care.

[92] Spallone V. (2019). Update on the impact, diagnosis and management of cardiovascular autonomic neuropathy in diabetes: What is defined, what is new, and what is unmet. Diabetes Metab. J..

[93] Warren B., Pankow J.S., Matsushita K., Punjabi N.M., Daya N.R., Grams M., Woodward M., Selvin E. (2017). Comparative prognostic performance of definitions of prediabetes: A prospective cohort analysis of the atherosclerosis risk in communities (ARIC) study. Lancet Diabetes Endocrinol..

[94] Bergman M., Abdul-Ghani M., DeFronzo R.A., Manco M., Sesti G., Fiorentino T.V., Ceriello A., Rhee M., Phillips L.S., Chung S. (2020). Review of methods for detecting glycemic disorders. Diabetes Res. Clin. Pract..

[95] Vaishya S., Sarwade R.D., Seshadri V. (2018). MicroRNA, proteins, and metabolites as novel biomarkers for prediabetes, diabetes, and related complications. Front. Endocrinol..

[96] Sidorkiewicz I., Niemira M., Maliszewska K., Erol A., Bielska A., Szałkowska A., Adamska-Patruno E., Szczerbinski L., Gorska M., Krętowski A. (2020). Circulating miRNAs as a predictive biomarker of the progression from prediabetes to diabetes: Outcomes of a 5-year prospective observational study. J. Clin. Med..

[97] Zhang J.R., Sun H.J. (2020). Roles of circular RNAs in diabetic complications: From molecular mechanisms to therapeutic potential. Gene.

[98] Li X., Zhao Z., Gao C., Rao L., Hao P., Jian D., Li W., Tang H., Li M. (2017). The diagnostic value of whole blood lncRNA ENST00000550337.1 for pre-diabetes and type 2 diabetes mellitus. Exp. Clin. Endocrinol. Diabetes.

[99] Zhang P., Zhu X., Du Y., Dong Z., Qiao C., Li T., Chen P., Lou P. (2020). Screening and functional studies of long noncoding RNA in subjects with prediabetes. Endocrine.

[100] Bridges K. (2013). Assessing Salivary Biomarkers for Risk Stratification in Metabolic Syndrome.

[101] Garg V., Kumar M., Mahapatra H.S., Chitkara A., Gadpayle A.K., Sekhar V. (2015). Novel urinary biomarkers in pre-diabetic nephropathy. Clin. Exp. Nephrol..

[102] Gu D., Chen Y., Masucci M., Xiong C., Zou H., Holthofer H. (2020). Potential urine biomarkers for the diagnosis of prediabetes and early diabetic nephropathy based on ISN CKHDP program. Clin. Nephrol..

[103] Li Y.P., Yan Z.Q., Han L.P., Yin A.L., Xu J.Y., Zhai Y.R., Hao S., Zhang L., Xie Y. (2020). The Association between phosphorylated neurofilament heavy chain (pNF-H) and small fiber neuropathy (SFN) in patients with impaired glucose tolerance. Diabetes Ther..

[104] Richdale K., Chao C., Hamilton M. (2020). Eye care providers’ emerging roles in early detection of diabetes and management of diabetic changes to the ocular surface: A review. BMJ Open Diabetes Res. Care.

[105] Greco M., Chiefari E., Accattato F., Corigliano D.M., Arcidiacono B., Mirabelli M., Liguori R., Brunetti F.S., Pullano S.A., Scorcia V. (2020). MicroRNA-1281 as a novel circulating biomarker in patients with diabetic retinopathy. Front. Endocrinol..

[106] American Diabetes Association 3 (2019). Prevention or delay of type 2 diabetes: Standards of medical care in diabetes—2020. Diabetes Care.

[107] Gabriel R., Abdelkader N.B., Acosta T., Gilis-Januszewska A., Gómez-Huelgas R., Makrilakis K., Kamenov Z., Paulweber B., Satman I., Djordjevic P. (2020). Early prevention of diabetes microvascular complications in people with hyperglycaemia in Europe. ePREDICE randomized trial. Study protocol, recruitment and selected baseline data. PLoS ONE.

[108] Papaetis G.S. (2014). Incretin-based therapies in prediabetes: Current evidence and future perspectives. World J. Diabetes.

[109] Wang X., Liu J., Huang L., Zeng H., He G., Chen L., Ma R., Fu W., Ning B. (2019). Anti-diabetic agents for prevention of type 2 diabetes mellitus in people with pre-diabetes: A systematic review and network meta-analysis protocol. BMJ Open.

[110] Luc K., Schramm-Luc A., Guzik T.J., Mikolajczyk T.P. (2019). Oxidative stress and inflammatory markers in prediabetes and diabetes. J. Physiol. Pharmacol..

[111] Huang Y.Q., Liu L., Huang C., Yu Y.L., Lo K., Huang J.Y., Chen C.L., Zhou Y.L., Feng Y.Q. (2020). Impacts of pre-diabetes or prehypertension on subsequent occurrence of cardiovascular and all-cause mortality among population without cardiovascular diseases. Diabetes Metab. Syndr. Obesity Targets Ther..

